# Trends in and Characteristics of Buprenorphine Misuse Among Adults in the US

**DOI:** 10.1001/jamanetworkopen.2021.29409

**Published:** 2021-10-15

**Authors:** Beth Han, Christopher M. Jones, Emily B. Einstein, Wilson M. Compton

**Affiliations:** 1 National Institute on Drug Abuse, National Institutes of Health, Bethesda, Maryland; 2 National Center for Injury Prevention and Control, Centers for Disease Control and Prevention, Atlanta, Georgia

## Abstract

**Importance:**

There is a lack of empirical research regarding misuse of buprenorphine hydrochloride.

**Objective:**

To identify prescription opioids that are most frequently misused, assess differences in motivations for misuse between buprenorphine and nonbuprenorphine prescription opioids, and examine trends in and factors associated with buprenorphine misuse among individuals with or without opioid use disorder (OUD).

**Design, Setting, and Participants:**

This survey study used nationally representative data on past-year prescription opioid use, misuse, OUD, and motivations for the most recent misuse from the 2015-2019 National Survey on Drug Use and Health (NSDUH). Participants included 214 505 civilian, noninstitutionalized adult NSDUH respondents. Data were collected from January 2015 to December 2019 and analyzed from February 15 to March 15, 2021.

**Main Outcomes and Measures:**

Buprenorphine use, misuse, and OUD. Misuse was defined as use “in any way that a doctor [physician] did not direct you to use them, including (1) use without a prescription of your own; (2) use in greater amounts, more often, or longer than you were told to take them; or (3) use in any other way a doctor did not direct you to use them.”

**Results:**

The 214 505 respondents included in the analysis represented an estimated annual average 246.7 million US adults during 2015-2019 (51.7% [95% CI, 51.4%-52.0%] women; 13.9% [95% CI, 13.7%-14.1%] aged 18-25 y; 40.6% [95% CI, 40.3%-41.0%] aged 26-49 y; 45.5% [95% CI, 45.0-45.9%] aged ≥50 y). In 2019, an estimated 2.4 (95% CI, 2.2-2.7) million US adults used buprenorphine, and an estimated 0.7 (95% CI, 0.5-0.9) million misused buprenorphine compared with an estimated 4.9 (95% CI, 4.4-5.4) million and an estimated 3.0 (95% CI, 2.7-3.2) million who misused hydrocodone and oxycodone, respectively. Prevalence of OUD with buprenorphine misuse trended downward during the period from 2015 to 2019. “Because I am hooked” (27.3% [95% CI, 21.6%-33.8%]) and “to relieve physical pain” (20.5% [95% CI, 14.0%-29.0%]) were the most common motivations for the most recent buprenorphine misuse among adults with OUD. Adults who misused buprenorphine were more likely to report using prescription opioids without having their own prescriptions than those who misused nonbuprenorphine prescription opioids (with OUD: 71.8% [95% CI, 66.4%-76.6%] vs 53.2% [95% CI, 48.5%-57.8%], *P* < .001; without OUD: 74.7% [95% CI, 68.7%-79.9%] vs 60.0% [58.1%-61.8%], *P* < .001). Among adults with past-year OUD who used buprenorphine, multivariable multinomial logistic regression results indicated that buprenorphine misuse was associated with being 24 to 34 (adjusted odds ratio [AOR], 2.9 [95% CI, 1.4-5.8]) and 35 to 49 (AOR, 2.3 [95% CI, 1.2-4.5]) years of age, residing in nonmetropolitan areas (AOR, 1.8 [95% CI, 1.0-3.0]), and polysubstance use (eg, past-year prescription stimulant use disorder; AOR, 3.9 [95% CI, 1.3-11.2]) but was negatively associated with receiving treatment for drug use only (AOR, 0.4 [95% CI, 0.3-0.7]).

**Conclusions and Relevance:**

These findings suggest that among adults with OUD, prevalence of buprenorphine misuse trended downward from 2015 to 2019. In 2019, nearly three-fourths of US adults reporting past-year buprenorphine use did not misuse their prescribed buprenorphine, and most who misused reported using prescription opioids without having their own prescriptions. These findings underscore the need to pursue actions that expand access to buprenorphine-based OUD treatment, to develop strategies to monitor and reduce buprenorphine misuse, and to address associated conditions (eg, suicide risk, co-occurring mental illness, and polysubstance use).

## Key Points

**Question:**

With recent increases in buprenorphine treatment for opioid use disorder (OUD), is buprenorphine misuse increasing in the US?

**Findings:**

In this survey study of 214 505 respondents to the National Survey on Drug Use and Health Data, nearly three-fourths of adults reporting buprenorphine use did not misuse their prescribed buprenorphine in 2019. Among adults with OUD, prevalence of buprenorphine misuse trended downward during the period from 2015 to 2019, and “because I am hooked” and “to relieve physical pain” were the most common motivations for the most recent buprenorphine misuse.

**Meaning:**

These findings underscore the need to expand access to buprenorphine-based OUD treatment while monitoring and implementing strategies to reduce buprenorphine misuse.

## Teaser

This survey study examines buprenorphine misuse among US adults with and without opioid use disorder.

## Introduction

Buprenorphine hydrochloride is a critical medication for treating opioid use disorder (OUD)^1,2,3^ and is prescribed to relieve severe pain for patients who need daily, continuous, long-term opioid treatment when other medications are inadequate.^4,5,6^ To prescribe buprenorphine for treatment of OUD, clinicians must obtain a waiver and are limited in the number of patients they can treat at one time. However, clinicians do not need a waiver to prescribe buprenorphine for pain management.

Multiple steps have been taken recently to expand access to buprenorphine-based OUD treatment (eg, expanding prescription authority to nonphysicians, raising the maximum patient limit to 275 for qualified clinicians, expanding insurance coverage). Although the number of clinicians receiving a waiver to prescribe buprenorphine for OUD has increased over time,^7,8,9^ only a small fraction of clinicians eligible to obtain a waiver have requested one, and an even smaller fraction actually prescribe buprenorphine. Concerns include unease with treating patients who have OUD, lack of adequate reimbursement, and risks for diversion, misuse, and overdose.^10,11,12,13,14,15,16^

On April 28, 2021, the US Department of Health and Human Services released practice guidelines for the administration of buprenorphine for treating OUD, aiming to increase OUD treatment, primarily by allowing a limited waiver for prescribing buprenorphine without the specialized training requirement.^17^ The exemption, specifically addressing reported barriers of the training requirement, allows licensed clinicians to (1) treat as many as 30 patients with OUD using buprenorphine without having to make certain training-related certifications and (2) treat patients with buprenorphine without certifying their capacity to provide counseling and ancillary services.^17^

Notably, buprenorphine treatment is complicated by concerns for misuse, defined as using buprenorphine without a prescription or without following a physician’s instructions.^18^ In particular, understanding the most frequently used and misused prescription opioids and the differences in the main motivations between buprenorphine misuse and other prescription opioid misuse can help address clinicians’ and policy makers’ concerns. Better understanding of buprenorphine use and misuse can inform policy and clinical practice development, education, training, and initiatives to expand access to this life-saving medication in a manner that is safe and minimizes harm.

To address these issues, we used nationally representative samples to examine the following:

1. Which prescription opioids are the most frequently misused by US adults?
2. Among US adults with buprenorphine use, has annual prevalence of buprenorphine misuse changed over time?
3. Among US adults who misuse prescription opioids, are there differences in the main motivations between the most recent buprenorphine misuse and nonbuprenorphine prescription opioid misuse?
4. Among US adults with buprenorphine use, what are sociodemographic characteristics, health conditions, and behavioral health factors associated with buprenorphine misuse?

Clinical and policy implications differ for persons with or without OUD who misuse buprenorphine. Moreover, because the primary use of buprenorphine is to treat OUD,^19,20^ because buprenorphine misuse and OUD are highly correlated, and because some people with OUD misuse nonprescribed buprenorphine to self-treat their OUD symptoms,^21,22,23,24^ we examined buprenorphine misuse among those with and without OUD as distinct categories.

## Methods

### Survey Methods and Study Population

We examined data from 214 505 adult respondents participating in the 2015-2019 National Survey on Drug Use and Health (NSDUH) conducted by the Substance Abuse and Mental Health Services Administration (data collection spanned from January 2015 to December 2019).^18,19,20,21,22,23,24,25^ With a protocol approved by the institutional review board at RTI International, NSDUH collected nationally representative data on past-year prescription opioid use, misuse, OUD, and motivations for the most recent misuse among US civilian, noninstitutionalized populations 12 years or older.^18,25,26^ Verbal informed consent was received from each participant. Race and ethnicity were determined according to NSDUH respondents’ self-classification of racial and ethnic origin and identification based on classifications developed by the US Census Bureau. For the 2015-2019 NSDUH, the annual mean weighted screening response rate was 75.3%, and the annual mean weighted interview response rate was 67.3% according to the reporting guideline for in-person household surveys by the American Association for Public Opinion Research (AAPOR).^27^

### Measures of Main Outcomes and Participant Characteristics

The 2015-2019 NSDUH asked about lifetime and past-year use and misuse of specific prescription opioids (eg, buprenorphine).^28^ The NSDUH defined prescription opioid misuse (including buprenorphine misuse) as use “in any way that a doctor [physician] did not direct you to use them, including (1) use without a prescription of your own; (2) use in greater amounts, more often, or longer than you were told to take them; or (3) use in any other way a doctor did not direct you to use them.”^18,25,26^ Any respondent meeting 1 of these 3 criteria would be classified as having buprenorphine misuse. For respondents with past-year prescription opioid misuse, NSDUH asked about using any prescription opioids without having their own prescriptions, the name of the prescription opioid most recently misused, and the main motivation for their most recent misuse, including the following: “to relieve physical pain,” “to relax or relieve tension,” “to experiment,” “to feel good or get high,” “to help with my feelings or emotions,” “to increase or decrease the effect(s) of other drugs,” or “because I am hooked.”

The NSDUH also collected lifetime and past-year use of tobacco, alcohol, cannabis, cocaine, heroin, inhalants, and hallucinogens and use and misuse of prescription stimulants and sedatives/tranquilizers. Using diagnostic criteria specified in the *Diagnostic and Statistical Manual of Mental Disorders, Fourth Edition*, *Text Revision* (*DSM-IV-TR*),^29^ the NSDUH estimated prevalence of past-year specific substance use disorders. Opioid use disorder was defined as prescription OUD or heroin use disorder and was assessed based on *DSM-IV-TR* criteria rather than self-classified status. The NSDUH assessed past-year major depressive episode using *DSM-IV-TR* criteria^29^ and past-month nicotine dependence among cigarette smokers using the Nicotine Dependence Syndrome Scale.^30^ These measures of substance use and substance use disorders have demonstrated good validity and reliability.^31,32,33^

In addition to sociodemographic characteristics, the NSDUH asked about medical diagnoses received from a physician or other health care professional (hypertension, heart disease, diabetes, cancer, chronic obstructive pulmonary disease, asthma, hepatitis B virus/hepatitis C virus), self-rated health, the number of past-year emergency department visits, and receipt of lifetime and past-year substance use treatment. The NSDUH asked adult respondents about past-year serious thoughts of suicide, suicide plan, and suicide attempt.

### Statistical Analysis

Data were analyzed from February 15 to March 15, 2021. First, we estimated the numbers of US adults in 2019 who used specific prescription opioid products in the past 12 months (misused and not misused). Second, among US adults reporting past-year buprenorphine use, we assessed trends^34^ in prevalence of OUD and buprenorphine misuse during the period from 2015 to 2019. Third, among adults reporting past-year prescription opioid misuse, we estimated past-year prevalence of using prescription opioids without having their own prescriptions and compared differences in the main motivation for the most recent buprenorphine misuse and nonbuprenorphine prescription opioid misuse by OUD status.

Fourth, among adults with past-year buprenorphine use, we examined differences in sociodemographic characteristics, health conditions, and behavioral health status between those with and without past-year buprenorphine misuse by OUD status at the bivariable level. To assess past-year buprenorphine use with and without misuse or OUD (4 outcomes) simultaneously, multivariable multinomial logistic regression modeling was applied. Multicollinearity and potential interaction effects were tested and were not found in final multinomial logistic regression models. All analyses used SUDAAN software^35^ to account for NSDUH’s complex sample design and sample weights. For all analyses, *P* < .05 (2-tailed) was considered statistically significant.

## Results

### Commonly Misused Prescription Opioids

**Figure 1.**
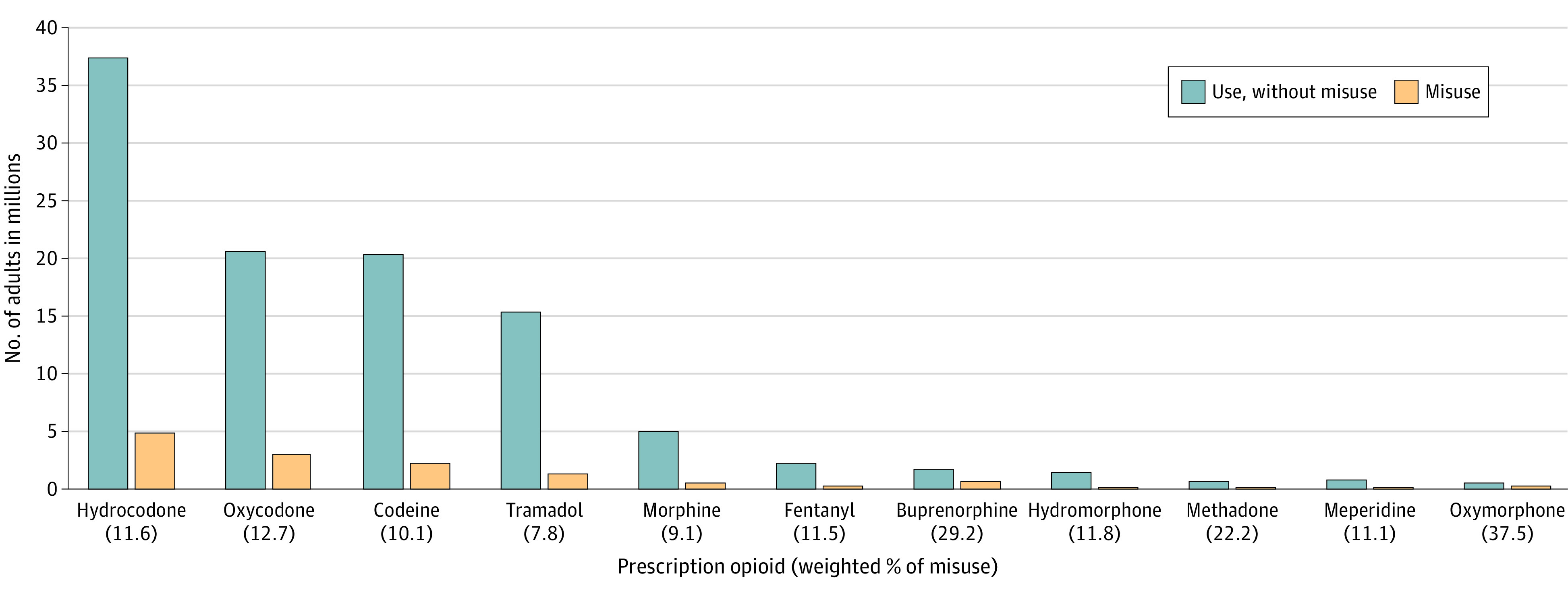
US Adults Who Reported Using or Misusing Prescription Opioids in the Past 12 Months Data are from 42 739 respondents in the 2019 National Survey on Drug Use and Health (NSDUH). The 2015-2019 NSDUH did not collect information on the use of illicitly manufactured fentanyl; the fentanyl data shown are for prescription fentanyl. For each type of prescription opioid, the denominator for estimating the proportion of misuse is the number of adults with use but no misuse plus the number of adults with misuse.

We examined data from 214 505 individuals 18 years or older representing an estimated annual average 246.7 million US adults during 2015-2019. Of these 246.7 million adults, 51.7% (95% CI, 51.4%-52.0%) were women and 48.3% (95% CI, 48.0%-48.6%) were men; 13.9% (95% CI, 13.7%-14.1%) were aged 18 to 25 years, 40.6% (95% CI, 40.3%-41.0%) were aged 26 to 49 years, and 45.5% (95% CI, 45.0-45.9%) were aged 50 years or older; and 16.0% (95% CI, 15.6%-16.4%) were Hispanic, 11.9% (95% CI, 11.5%-12.2%) were non-Hispanic Black, and 63.9% (95% CI, 63.3%-64.4%) were non-Hispanic White. In 2019, the prescription opioid products most commonly misused during the past 12 months by US adults were hydrocodone (estimated 4.9 [95% CI, 4.4-5.4] million people), oxycodone (estimated 3.0 [95% CI, 2.7-3.2] million people), codeine (estimated 2.3 [95% CI, 2.0-2.5] million people), and tramadol (estimated 1.3 [95% CI, 1.1-1.5] million people) (Figure 1). Among US adults in 2019, an estimated 2.4 (95% CI, 2.2-2.7) million used buprenorphine and an estimated 0.7 (95% CI, 0.5-0.9) million misused buprenorphine in the past 12 months, whereas an estimated 1.7 (95% CI, 1.5-1.9) million used buprenorphine without misuse.

### Trends in OUD and Buprenorphine Misuse

**Figure 2.**
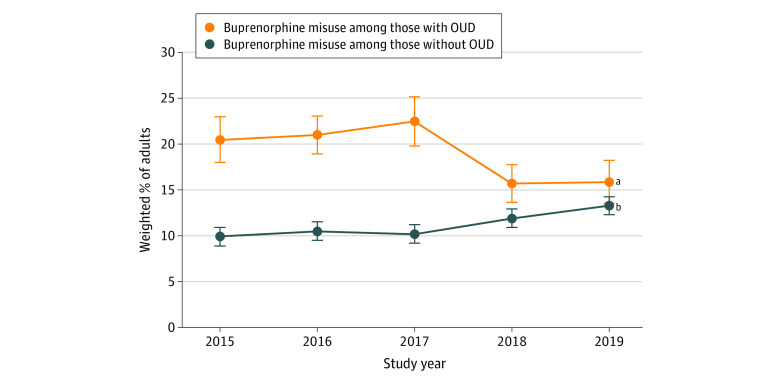
Trends in Prevalence of Past-Year Buprenorphine Misuse by Opioid Use Disorder (OUD) Status Among US Adults With Past-Year Buprenorphine Use Data are from 2536 respondents in the 2015-2019 National Surveys on Drug Use and Health. Error bars indicate SEs. ^a^Linear trend: *P* = .04. ^b^Linear trend: *P* = .08.

For US adults who used buprenorphine in the past year, the prevalence of buprenorphine misuse remained stable among individuals without OUD; among those with OUD, buprenorphine misuse in each year from 2015 to 2018 did not differ significantly from 2019, but the overall trend was downward (Figure 2). In 2019, among adults with past-year buprenorphine use, 29.2% (95% CI, 23.7%-35.4%) misused buprenorphine; specifically, 13.3% [95% CI, 9.7%-17.9%) had buprenorphine misuse but no OUD, 15.9% (95% CI, 11.8%-21.2%) had buprenorphine misuse and OUD, and 61.0% (95% CI, 54.3%-67.3%) had neither buprenorphine misuse nor OUD.

### Past-Year Prevalence of Using Any Prescription Opioid Without a Prescription

Among US adults with past-year prescription opioid misuse, prevalence of using any prescription opioid (including buprenorphine) without having their own prescriptions at any time during the past 12 months was higher among those with buprenorphine misuse than among those with nonbuprenorphine prescription opioid misuse regardless of OUD status (with OUD: 71.8% [95% CI, 66.4%-76.6%] vs 53.2% [95% CI, 48.5%-57.8%], *P* < .001; without OUD: 74.7% [95% CI, 68.7%-79.9%] vs 60.0% [95% CI, 68.7%-79.9%], *P* < .001).

### Motivations for Prescription Opioid Misuse

Table 1 shows differences in the main motivation between the most recent buprenorphine misuse and nonbuprenorphine prescription opioid misuse among adults with past-year prescription opioid misuse. “Because I am hooked” (27.3% [95% CI, 21.6%-33.8%]) and “to relieve physical pain” (20.5% [95% CI, 14.0%-29.0%]) were the most common motivations for the most recent buprenorphine misuse among adults with OUD, whereas “to relieve physical pain” (29.3% [95% CI, 21.2%-39.1%]) and “to feel good or get high” (18.1% [95% CI, 11.5%-27.4%]) were the most common motivations for the most recent buprenorphine misuse among adults without OUD. Among adults with OUD, compared with those with nonbuprenorphine prescription opioid misuse, those with buprenorphine as their most recent opioid misuse were more likely to report “because I am hooked” (27.3% [95% CI, 21.6%-33.8%] vs 7.8% [95% CI, 6.2%-9.9%]) and “to increase/decrease effects of other drug(s)” (15.1% [95% CI, 9.5%-23.1%] vs 1.2% [95% CI, 0.3%-4.4%]), but were less likely to report “to relieve physical pain” (20.5% [95% CI, 14.0%-29.0%] vs 52.2% [95% CI, 47.6%-56.8%]), “to relax or relieve tension” (3.7% [95% CI, 1.6%-8.3%] vs 8.9% [95% CI, 7.0%-11.1%]), and “to feel good or get high” (9.4% [95% CI, 5.9%-14.6%] vs 17.1% [95% CI, 14.4%-20.2%]) as their main motivations.

**Table 1.** Differences in Main Motivation Between the Most Recent Buprenorphine Misuse and Nonbuprenorphine Prescription Opioid Misuse by Past-Year Buprenorphine Misuse and OUD Status

Main motivation for misuse	OUD status, weighted % (95% CI)^a^			
	OUD		No OUD	
	Nonbuprenorphine prescription misuse (n = 1382)	Buprenorphine misuse (n = 233)	Nonbuprenorphine prescription misuse (n = 7898)	Buprenorphine misuse (n = 213)
Relieve physical pain	52.2 (47.6-56.8)^b^	20.5 (14.0-29.0)^b^^,^^c^	66.6 (65.0-68.2)^c^	29.3 (21.2-39.1)^b^^,^^c^
Relax or relieve tension	8.9 (7.0-11.1)	3.7 (1.6-8.3)^b^^,^^c^	10.5 (9.4-11.7)	6.8 (3.2-14.0)
Experiment	1.4 (0.7-2.7)	1.6 (0.5-5.4)	2.4 (2.1-2.8)	8.5 (4.9-14.3)^b^^,^^c^
Feel good or get high	17.1 (14.4-20.2)^b^	9.4 (5.9-14.6)^c^	10.3 (9.4-11.3)^c^	18.1 (11.5-27.4)^b^
Help with my feelings or emotions	6.3 (4.8-8.2)^b^	8.2 (3.4-18.6)^b^^,^^d^	2.6 (2.1-3.2)^c^	11.7 (5.8-22.2)^b^^,^^d^
Increase/decrease effect(s) of other drugs	1.2 (0.3-4.4)^d^	15.1 (9.5-23.1)^b^^,^^c^	0.5 (0.3-0.8)	3.6 (2.2-5.9)^b^
Because I am hooked	7.8 (6.2-9.9)^b^	27.3 (21.6-33.8)^b^^,^^c^	0.2 (0.1-0.3)^c^	12.7 (7.3-21.2)^b^

Abbreviation: OUD, opioid use disorder.

^a^ Data are from 9726 respondents in the 2015-2019 National Surveys on Drug Use and Health.

^b^ This estimate is statistically significantly (*P* < .05) different from the estimate of the corresponding adults with nonbuprenorphine prescription opioid misuse but no OUD (within each row).

^c^ This estimate is statistically significantly (*P* < .05) different from the estimate of the corresponding adults with nonbuprenorphine opioid misuse and with OUD (within each row).

^d^ Interpret with caution owing to low statistical precision.

### Differences in Sociodemographic Characteristics, Health Conditions, and Behavioral Health Status by OUD and Buprenorphine Misuse

Among adults with past-year buprenorphine use and OUD (Table 2), those with buprenorphine misuse were more likely to be non-Hispanic White individuals (82.9% [SE, 2.2%] vs 73.6% [SE, 4.1%]) and less likely to reside in large metropolitan areas (47.7% [SE, 3.1%] vs 58.1% [SE, 3.3%]), but those with and without buprenorphine misuse were at similarly high risk for having a suicide plan (8.2% [SE, 1.7%] and 12.3% [SE, 2.3%], respectively). They were also more likely to have polysubstance use and use disorders (eg, past-year prescription stimulant misuse or use disorder; 36.7% [SE, 2.9%] vs 15.0% [SE, 2.0%]) and were less likely to receive treatment for illicit drug use only (23.5% [SE, 2.1%] vs 37.8% [SE, 3.8%]) in the past year. Among adults with buprenorphine use but without OUD, adults with buprenorphine misuse were more likely to have past-year major depressive episode (21.5% [SE, 3.0%] vs 15.0% [1.3%]), suicidality (eg, suicide plan; 11.0% [SE, 2.8%] vs 2.0% [SE, 0.5%]), and polysubstance use and use disorders (eg, past-year prescription stimulant misuse or use disorder; 30.8% [SE, 3.4%] vs 7.2% [SE, 0.7%]) and were less likely to receive drug use–only treatment (6.6% [SE, 1.3%] vs 13.0% [SE, 1.1%]) in the past year.

**Table 2.** Differences in Sociodemographic Characteristics, Health Conditions, and Behavioral Health Status by Past-Year OUD and Buprenorphine Misuse Status Among US Adults With Past-Year Buprenorphine Use

Characteristic	Misuse status, weighted % (SE)^a^					
	Overall		OUD			
			Yes		No	
	No misuse (n = 1617)	Misuse (n = 919)	No misuse (n = 360)	Misuse (n = 558)	No misuse (n = 1257)	Misuse (n = 361)
Age, y						
18-23	7.7 (0.5)	13.2 (1.2)^b^	8.5 (1.3)	11.6 (1.3)	7.5 (0.5)	16.0 (2.0)^b^
24-34	29.7 (1.3)	44.9 (2.2)^b^	37.9 (3.7)	46.9 (2.5)	27.5 (1.6)	41.4 (3.7)^b^
35-49	29.3 (1.5)	28.1 (1.6)	30.5 (4.0)	29.6 (2.1)	29.0 (1.5)	25.5 (3.1)
≥50	33.2 (2.0)	13.8 (2.2)^b^	23.1 (5.6)	11.9 (2.4)^b^	36.0 (2.3)	17.1 (3.6)^b^
Sex						
Men	54.0 (1.6)	61.5 (2.1)^b^	62.0 (3.1)	62.6 (2.5)	51.8 (1.8)	59.7 (4.1)
Women	46.0 (1.6)	38.5 (2.1)^b^	38.0 (3.1)	37.4 (2.5)	48.2 (1.8)	40.3 (4.1)
Race and ethnicity^c^						
Hispanic	15.5 (1.8)	9.3 (1.4)^b^	12.6 (3.2)	7.7 (1.4)	16.3 (2.1)	12.0 (2.7)
Non-Hispanic						
Black	9.3 (1.2)	6.4 (1.4)	9.7 (3.2)	6.7 (1.7)	9.2 (1.3)	5.8 (1.9)
White	70.2 (1.8)	81.0 (2.0)^b^	73.6 (4.1)	82.9 (2.2)^b^	69.2 (2.1)	77.7 (3.5)^b^
Non-Hispanic other	5.1 (0.9)	3.4 (0.5)	4.1 (1.4)	2.7 (0.6)	5.4 (1.1)	4.5 (1.2)
Educational attainment						
Less than high school	21.7 (1.4)	18.9 (2.1)	17.7 (2.5)	18.4 (2.5)	22.9 (1.8)	19.8 (2.8)
High school	30.8 (1.7)	37.4 (2.7)^b^	32.8 (4.4)	37.8 (3.0)	30.2 (1.7)	36.7 (3.7)
Some college	34.0 (1.9)	32.6 (2.0)	36.2 (4.0)	32.4 (2.6)	33.4 (2.0)	33.0 (3.8)
College graduate	13.5 (1.6)	11.1 (1.5)	13.3 (3.0)	11.5 (2.0)	13.6 (1.7)	10.5 (2.7)
Health insurance						
Private only	31.5 (1.8)	29.2 (2.1)	27.8 (2.9)	28.8 (2.7)	32.5 (2.2)	29.9 (3.3)
Uninsured	14.5 (1.3)	21.7 (1.8)^b^	18.4 (3.5)	21.8 (2.3)	13.5 (1.2)	21.6 (2.7)^b^
Medicaid only	28.1 (1.4)	36.0 (2.7)^b^	35.0 (3.5)	38.9 (3.5)	26.1 (1.6)	31.0 (2.7)
Other	25.9 (1.6)	13.1 (1.7)^b^	18.7 (3.3)	10.5 (1.5)^b^	27.9 (1.9)	17.5 (2.7)^b^
Marital status						
Married	32.8 (1.9)	19.0 (2.0)^b^	24.7 (3.8)	16.7 (2.4)	35.0 (1.9)	23.0 (3.5)^b^
Widowed	6.9 (1.0)	1.8 (0.8)^b^	2.8 (2.2)^d^	1.9 (1.1)^d^	8.0 (1.2)	1.8 (1.4)^d^
Divorced/separated	20.3 (1.4)	20.3 (1.9)	21.4 (3.3)	19.1 (2.4)	19.9 (1.7)	22.3 (3.5)
Never married	40.1 (1.8)	58.9 (2.3)^b^	51.1 (4.8)	62.4 (3.1)	37.1 (1.9)	52.9 (47.0)^b^
Employment status						
Full-time	34.5 (1.9)	40.8 (2.2)^b^	39.6 (4.2)	40.6 (3.2)	33.1 (2.1)	41.1 (2.8)
Part-time	10.4 (1.0)	12.0 (1.7)	8.4 (1.50)	12.6 (2.4)	10.9 (1.2)	10.9 (2.4)
Unemployment	14.3 (1.4)	16.1 (1.7)	16.5 (2.5)	17.2 (2.1)	13.7 (1.6)	14.3 (2.1)
Other	40.9 (2.0)	31.1 (2.4)^b^	35.6 (4.7)	29.6 (2.8)	42.4 (2.1)	33.7 (3.9)
Family income, $						
<20 000	29.9 (1.7)	32.7 (1.9)	33.9 (3.4)	30.0 (2.8)	28.8 (2.1)	37.1 (3.3)
20 000-49 999	34.9 (1.7)	34.2 (2.2)	31.4 (3.5)	32.5 (2.8)	35.9 (1.9)	37.1 (3.3)
50 000-74 999	13.3 (1.1)	14.0 (1.9)	9.8 (1.8)	15.4 (2.5)	14.3 (1.3)	11.7 (2.4)
≥75 000	21.9 (1.7)	19.1 (1.6)	24.9 (3.5)	22.1 (2.4)	21.1 (1.8)	14.1 (2.1)
Metropolitan statistical area						
Large	50.7 (1.8)	44.3 (2.3)^b^	58.1 (3.3)	47.7 (3.1)^b^	48.7 (2.2)	38.6 (3.9)^b^
Small	34.2 (1.6)	36.1 (2.3)	28.9 (3.4)	34.3 (3.0)	35.6 (1.8)	39.3 (3.9)
None	15.1 (1.3)	19.5 (1.6)	13.0 (2.2)	18.1 (1.9)	15.7 (1.6)	22.1 (2.6)^b^
Self-rated health						
Excellent	9.9 (1.3)	7.9 (1.2)	6.1 (1.9)	5.0 (1.0)	10.8 (1.5)	12.8 (2.7)
Very good	24.8 (1.3)	29.7 (2.0)^b^	24.6 (3.3)	29.9 (3.1)	24.9 (1.4)	29.5 (3.7)
Good	33.9 (1.8)	36.1 (2.8)	41.1 (4.1)	38.7 (3.3)	31.9 (1.9)	31.5 (3.1)
Fair/poor	31.5 (1.9)	26.3 (2.5)	28.2 (3.7)	26.4 (3.2)	32.5 (2.1)	26.2 (3.7)
No. of past-year ED visits						
0	52.8 (1.7)	52.3 (2.2)	47.0 (3.8)	50.9 (2.7)	54.4 (2.0)	54.8 (4.2)
1	19.8 (1.4)	18.6 (1.6)	21.4 (3.1)	19.2 (1.7)	19.3 (1.7)	17.5 (2.8)
2	15.8 (1.7)	15.1 (1.7)	14.0 (2.4)	14.1 (2.1)	16.3 (1.9)	16.7 (2.9)
≥3	11.6 (1.1)	14.0 (1.5)	17.7 (3.2)	15.9 (2.3)	9.9 (1.1)	10.9 (2.1)
Hypertension	14.1 (1.4)	12.6 (1.7)	11.7 (2.3)	11.2 (2.0)	14.7 (1.6)	15.1 (3.1)
Heart disease	11.2 (1.4)	7.6 (1.4)	8.3 (1.8)	7.1 (4.5)	12.0 (1.7)	8.5 (2.9)
Diabetes	9.7 (1.1)	7.1 (1.5)	6.0 (1.5)	6.6 (1.9)	10.7 (1.2)	8.1 (2.3)
Cancer	4.5 (0.8)	3.6 (1.2)	5.8 (1.8)	3.0 (1.4)	4.1 (0.9)	4.7 (2.3)
Asthma	11.8 (1.4)	9.5 (1.4)	12.0 (2.4)	10.1 (1.9)	11.7 (1.5)	8.4 (1.7)
COPD	7.0 (1.0)	6.2 (1.1)	6.0 (1.6)	8.0 (1.7)	7.2 (1.1)	2.9 (1.0)^b^
HBV/HCV	8.3 (0.9)	11.3 (1.4)	12.6 (2.6)	13.3 (1.9)	7.3 (1.0)	7.8 (1.8)
Major depressive episode	16.6 (1.2)	26.8 (2.0)^b^	22.2 (3.2)	29.9 (2.7)	15.0 (1.3)	21.5 (3.0)^b^
Suicide ideation	10.9 (1.0)	20.3 (1.8)^b^	22.0 (2.3)	20.3 (2.1)	7.8 (1.1)	20.2 (3.5)^b^
Past-year suicide plan	4.2 (0.7)	9.2 (1.4)^b^	12.3 (2.3)	8.2 (1.7)	2.0 (0.5)	11.0 (2.8)^b^
Suicide attempt	2.7 (0.4)	4.6 (0.8)^b^	7.1 (1.8)	5.4 (1.0)	1.5 (0.4)	3.3 (1.2)^b^
Past-year mental health care	34.9 (1.6)	41.6 (2.1)^b^	41.8 (4.3)	46.0 (2.6)	33.0 (2.0)	33.8 (3.6)
Tobacco						
Past-month nicotine dependence	43.3 (2.0)	66.9 (1.9)^b^	60.3 (5.0)	72.0 (2.4)^b^	38.6 (2.2)	58.3 (3.6)^b^
Past-year use, no past-month nicotine dependence	21.5 (1.6)	22.0 (1.8)	26.0 (4.2)	21.0 (2.3)	20.3 (1.6)	23.8 (2.9)
No past-year use	35.2 (2.4)	11.1 (1.4)^b^	13.7 (2.8)	7.1 (1.1)^b^	41.1 (2.7)	18.0 (3.4)^b^
Alcohol						
Past-year use disorder	11.9 (1.4)	25.9 (1.5)^b^	18.8 (3.5)	26.3 (2.3)	10.0 (1.5)	25.2 (2.9)^b^
Past-year use but no disorder	51.7 (1.7)	56.2 (2.0)	50.6 (4.1)	55.0 (2.6)	52.0 (2.2)	58.2 (3.1)
No past-year use	36.4 (1.6)	17.9 (1.7)^b^	30.6 (2.9)	18.7 (2.1)^b^	38.0 (2.1)	16.6 (2.6)^b^
Cannabis						
Past-year use disorder	4.9 (0.6)	10.7 (1.5)^b^	10.1 (2.1)	13.6 (2.1)	3.4 (0.6)	5.8 (1.3)
Past-year use but no use disorder	29.9 (1.7)	57.1 (2.0)^b^	43.5 (4.3)	57.9 (2.9)	26.1 (1.7)	55.7 (3.2)
Lifetime use, no past-year use	43.3 (2.2)	28.3 (1.8)^b^	43.4 (4.5)	27.2 (2.7)^b^	43.6 (2.5)	30.2 (3.2)^b^
Never used	21.9 (1.9)	3.9 (1.1)^b^	4.0 (1.3)	1.4 (0.6)	26.9 (2.2)	8.3 (2.3)^b^
Cocaine						
Past-year use disorder	3.3 (0.4)	14.7 (1.7)^b^	11.0 (1.7)	19.9 (2.4)^b^	1.2 (0.3)	6.0 (2.0)^b^
Past-year use but no disorder	9.3 (1.1)	22.5 (1.7)^b^	18.5 (3.6)	23.4 (2.3)	6.7 (1.0)	20.8 (3.2)^b^
Lifetime use but no past-year use	43.9 (1.5)	49.2 (2.6)	51.6 (3.9)	48.3 (2.9)	41.8 (1.7)	50.7 (4.6)^b^
Never used	43.5 (1.8)	13.6 (1.5)^b^	18.9 (3.5)	8.4 (1.2)^b^	50.3 (1.8)	22.5 (3.1)^b^
Heroin						
Past-year use or use disorder	13.4 (1.4)	36.8 (2.5)^b^	51.4 (3.7)	52.1 (2.6)	2.9 (0.5)	10.9 (2.2)^b^
Lifetime use but no past-year use	21.5 (1.5)	22.5 (1.7)	13.9 (2.4)	19.1 (2.0)	23.6 (1.8)	28.3 (3.3)
Never used	65.1 (1.7)	40.6 (2.2)^b^	34.7 (3.8)	28.8 (2.6)	73.5 (1.8)	60.8 (3.6)^b^
Hallucinogen						
Past-year use or disorder	5.8 (0.7)	20.2 (1.7)^b^	10.7 (1.7)	19.8 (2.2)^b^	4.4 (0.7)	20.9 (3.0)^b^
Lifetime use but no past-year use	48.1 (1.8)	60.6 (2.0)^b^	61.9 (3.8)	64.8 (2.5)	44.3 (2.0)	53.3 (3.8)^b^
Never used	46.1 (1.9)	19.2 (1.9)^b^	27.4 (3.5)	15.4 (2.0)^b^	51.3 (2.0)	25.7 (3.5)^b^
Inhalant						
Past-year use or use disorder	2.1 (0.5)	6.0 (0.9)^b^	2.7 (1.0)	6.1 (1.2)^b^	2.0 (0.6)	5.8 (1.1)^b^
Lifetime use but no past-year use	30.5 (1.7)	46.4 (2.7)^b^	45.1 (4.5)	47.9 (3.7)	26.5 (1.9)	44.0 (4.0)^b^
Never used	67.4 (1.7)	47.6 (2.7)^b^	52.2 (4.3)	46.1 (3.7)	71.5 (1.8)	50.2 (3.8)^b^
Prescription sedative/tranquilizer						
Past-year misuse or use disorder	17.2 (1.3)	52.7 (2.3)^b^	38.2 (3.8)	59.5 (2.5)^b^	11.4 (1.3)	41.4 (3.5)^b^
Past-year use, lifetime misuse	5.8 (0.8)	6.3 (1.1)	10.3 (2.3)	6.9 (1.5)	4.5 (0.8)	5.3 (1.4)
Past-year use, no lifetime misuse	32.9 (1.7)	9.7 (1.3)^b^	25.2 (3.5)	9.3 (1.7)^b^	35.0 (2.1)	10.3 (1.9)^b^
Lifetime use but no past-year use	9.5 (0.9)	5.8 (1.0)^b^	6.1 (1.1)	4.2 (0.8)	10.4 (1.3)	8.6 (2.3)
Never used	34.7 (1.4)	25.5 (2.2)^b^	20.2 (3.7)	20.2 (2.3)	38.8 (1.7)	34.4 (3.4)
Prescription stimulant						
Past-year misuse or use disorder	8.9 (0.9)	34.5 (2.3)^b^	15.0 (2.0)	36.7 (2.9)^b^	7.2 (0.7)	30.8 (3.4)^b^
Past-year use, lifetime misuse	3.1 (0.6)	6.3 (1.0)^b^	5.3 (1.7)	6.6 (1.5)	2.4 (0.6)	5.9 (1.4)^b^
Past-year use, no lifetime misuse	15.5 (1.3)	7.8 (1.1)^b^	16.2 (3.2)	9.2 (1.6)^b^	15.3 (1.3)	5.5 (1.4)^b^
Lifetime use but no past-year use	7.9 (0.9)	4.5 (0.9)	11.2 (3.0)	4.7 (1.1)^b^	7.0 (0.9)	6.8 (1.8)
Never used	64.7 (1.6)	45.9 (2.8)^b^	52.3 (4.5)	42.9 (3.4)	68.1 (1.7)	51.0 (3.8)^b^
Product misuse						
Hydrocodone	19.1 (1.5)	57.0 (2.7)^b^	55.2 (4.4)	61.6 (3.0)	9.1 (1.2)	49.1 (3.8)^b^
Oxycodone	16.1 (1.3)	61.2 (2.7)^b^	49.5 (3.9)	70.6 (2.5)^b^	6.8 (1.0)	45.2 (4.2)^b^
Tramadol	5.0 (0.8)	19.6 (1.9)^b^	14.7 (2.7)	22.8 (2.8)	2.3 (0.7)	14.1 (2.5)^b^
Codeine	3.7 (0.8)	14.6 (1.8)^b^	10.9 (3.0)	18.0 (2.6)	1.7 (0.6)	8.9 (1.8)^b^
Morphine	4.7 (0.7)	21.0 (1.9)^b^	17.5 (2.5)	25.8 (2.2)	1.2 (0.4)	12.9 (2.8)^b^
Fentanyl	3.5 (0.6)	15.5 (1.6)^b^	11.6 (2.0)	20.6 (2.2)^b^	1.3 (0.5)	6.7 (2.1)^b^
Oxymorphone	2.8 (0.5)	18.5 (1.6)^b^	8.7 (1.7)	24.0 (2.1)^b^	1.2 (0.5)	9.2 (2.1)^b^
Methadone	2.4 (0.7)	16.2 (1.7)^b^	7.7 (3.0)	22.3 (2.5)^b^	0.9 (0.3)	5.9 (1.8)^b^
Past-year substance use treatment						
Drug only	18.4 (1.2)	17.2 (1.4)	37.8 (3.8)	23.5 (2.1)^b^	13.0 (1.1)	6.6 (1.3)^b^
Alcohol and drug	5.5 (0.8)	14.3 (1.5)^b^	13.2 (3.0)	19.2 (2.3)	3.43 (0.7)	6.1 (1.6)
None	76.1 (1.4)	68.5 (1.9)^b^	49.0 (4.4)	57.3 (3.0)	83.5 (1.3)	87.3 (2.0)

Abbreviations: ED, emergency department; COPD, chronic obstructive pulmonary disorder; HBV/HCV, hepatitis B virus/hepatitis C virus; OUD, opioid use disorder.

^a^ Data are from 2536 respondents to the 2015-2019 National Surveys on Drug Use and Health (NSDUH).

^b^ Each estimate is significantly (*P* < .05) different from the estimate of the corresponding group with no buprenorphine misuse (within each major column and within each row).

^c^ Determined according to NSDUH respondents’ self-classification of racial and ethnic origin and identification based on the classifications developed by the US Census Bureau.

^d^ Interpret with caution owing to low statistical precision.

### Factors Associated With Buprenorphine Misuse and OUD

Multivariable multinomial logistic regression results (Table 3 and eTable in the Supplement) indicate that among adults with buprenorphine use and OUD, buprenorphine misuse was associated with being 24 to 34 (adjusted odds ratio [AOR], 2.9 [95% CI, 1.4-5.8]) and 35 to 49 (AOR, 2.3 [95% CI, 1.2-4.5]) years of age, residing in nonmetropolitan areas (AOR, 1.8 [95% CI, 1.0-3.0]), and having past-year polysubstance use and use disorders (eg, past-year prescription stimulant use disorder; AOR, 3.9 [95% CI, 1.3-11.2]) but was negatively associated with past-year treatment for illicit drug use–only treatment (AOR, 0.4 [95% CI, 0.3-0.7]). Among adults with buprenorphine use but without OUD, buprenorphine misuse was associated with being 24 to 34 years of age (AOR, 2.1 [95% CI, 1.2-4.2]) and having a past-year family income of less than $20 000 (AOR, 1.9 [95% CI, 1.1-3.3]), a suicide plan (AOR, 4.1 [95% CI, 1.7-9.8]), and polysubstance use and use disorders (eg, past-year cocaine use or use disorder; AOR, 4.0 [95% CI, 2.3-6.9]) but was negatively associated with drug use–only treatment (AOR, 0.4 [95% CI, 0.2-0.6]).

**Table 3.** Multivariable Multinomial Logistic Regression Factors Associated With Past-Year OUD and Buprenorphine Misuse Status Among US Adults With Past-Year Buprenorphine Use

Characteristic	Misuse status, AOR (95% CI)^a^	
	OUD: Misuse vs no misuse	No OUD: Misuse vs no misuse
Age, y		
18-23	2.0 (0.8-5.0)	2.0 (0.9-4.8)
24-34	2.9 (1.4-5.8)^b^	2.1 (1.1-4.2)^b^
35-49	2.3 (1.2-4.5)^b^	1.6 (0.9-2.8)
≥50	1 [Reference]	1 [Reference]
Sex		
Men	0.8 (0.5-1.3)	1.1 (0.7-1.7)
Women	1 [Reference]	1 [Reference]
Race and ethnicity		
Hispanic	0.7 (0.4-1.5)	1.3 (0.6-2.6)
Non-Hispanic		
Black	1.5 (0.8-2.8)	1.0 (0.4-2.6)
White	1 [Reference]	1 [Reference]
Non-Hispanic other	0.9 (0.4-1.9)	1.2 (0.5-2.7)
Family income, $		
<20 000	1.1 (0.6-1.9)	1.9 (1.1-3.3)^b^
20 000-49 999	1.1 (0.6-1.8)	1.3 (0.8-2.3)
50 000-74 999	2.0 (0.9-4.3)	1.2 (0.6-2.4)
≥75 000	1 [Reference]	1 [Reference]
Metropolitan statistical area		
Large	1 [Reference]	1 [Reference]
Small	1.5 (1.0-2.2)	1.4 (0.9-2.1)
None	1.8 (1.0-3.0)^b^	1.4 (0.9-2.4)
Suicide plan		
Yes	0.5 (0.2-1.1)	4.1 (1.7-9.8)^b^
No	1 [Reference]	1 [Reference]
Tobacco		
Past-month nicotine dependence	1.2 (0.6-2.4)	1.4 (0.7-2.6)
Past-year use, no past-month nicotine dependence	0.9 (0.5-1.8)	1.0 (0.6-2.0)
No past-year use	1 [Reference]	1 [Reference]
Alcohol		
Past-year use disorder	1.6 (0.9-2.7)	1.8 (1.0-3.2)^b^
Past-year use but no disorder	1.5 (1.0-2.3)	1.7 (1.1-2.9)^b^
No past-year use	1 [Reference]	1 [Reference]
Cannabis		
Past-year use disorder	1.4 (0.7-3.1)	0.8 (0.4-1.5)
Past-year use but no disorder	1.2 (0.8-1.9)	1.5 (1.0-2.2)^b^
No past-year use	1 [Reference]	1 [Reference]
Cocaine		
Past-year use or disorder	2.3 (1.1-4.7)^b^	4.0 (2.3-6.9)^b^
Lifetime use but no past-year use	1.9 (1.0-3.6)^b^	2.1 (1.4-3.3)^b^
Never used	1 [Reference]	1 [Reference]
Prescription sedative/tranquilizer		
Past-year misuse and disorder	0.9 (0.5-1.7)	0.9 (0.6-1.5)
Past-year use but no misuse	0.3 (0.2-0.6)^b^	0.3 (0.2-0.5)^b^
Lifetime use but no past-year use	0.6 (0.3-1.3)	0.7 (0.3-1.5)
Never used	1 [Reference]	1 [Reference]
Prescription stimulant		
Past-year use disorder	3.9 (1.3-11.2)^b^	3.3 (0.9-12.3)
Past-year misuse but no disorder	1.6 (1.0-2.6)	1.2 (0.7-2.2)
No past-year misuse	1 [Reference]	1 [Reference]
Past-year hydrocodone misuse		
Yes	0.8 (0.5-1.3)	4.3 (2.7-7.1)^b^
No	1 [Reference]	1 [Reference]
Past-year oxycodone misuse		
Yes	1.9 (1.2-3.1)^b^	3.9 (2.3-6.5)^b^
No	1 [Reference]	1 [Reference]
Past-year substance use treatment		
Drug only	0.4 (0.3-0.7)^b^	0.4 (0.2-0.6)^b^
Alcohol and drug	1.0 (0.6-1.9)	0.7 (0.3-1.6)
None	1 [Reference]	1 [Reference]

Abbreviations: AOR, adjusted odds ratio; OUD, opioid use disorder.

^a^ Data are from 2469 respondents to the 2015-2019 National Surveys on Drug Use and Health.

^b^ Each estimate is significantly (*P* < .05) different from the estimate of the reference group. Other variables, which were presented in Table 2 but not in Table 3, were not significantly associated with the outcomes and were removed from this final multinomial logistic regression model. Age, sex, and race and ethnicity remained in the final model regardless of their statistical significance. All multinomial logistic regression results are provided in the eTable in the Supplement.

## Discussion

Despite recent increases in buprenorphine treatment for OUD in the US,^10^ during the period from 2015 to 2019, prevalence of buprenorphine misuse without OUD remained stable, and prevalence of buprenorphine misuse with OUD trended downward. In 2019, hydrocodone and oxycodone were much more commonly misused (by 4.9 million and 3.0 million adults, respectively). By contrast, among 2.4 million US adults reporting past-year buprenorphine use, 0.7 million (or 29.2%) misused it, suggesting that almost three-fourths of adults reporting buprenorphine use in the past 12 months did not misuse their prescribed buprenorphine. Notably, among adults with past-year prescription opioid misuse, using prescription opioids without having their own prescriptions was more frequent among those who misused buprenorphine (71.8%-74.7%) than those who misused other prescription opioids (53.2%-60.0%), regardless of OUD status, suggesting that diversion from other persons is particularly common among adults with buprenorphine misuse. We also found that “because I am hooked” (27.3%) for self-treatment of craving and withdrawal symptoms and “to relieve physical pain” (20.5%) were the most common motivations for the most recent buprenorphine misuse among adults with OUD, whereas “to relieve physical pain” (29.3%) and “to feel good or get high” (18.1%) were the most common motivations for the most recent buprenorphine misuse among adults without OUD. Taken together, our results highlight the urgent need to expand access to buprenorphine-based OUD treatment and improve pain management while developing strategies to monitor and reduce buprenorphine misuse.

Although opioid treatment outcomes exhibit vast disparities, our multivariable results indicate that race and ethnicity and health insurance status were not associated with buprenorphine misuse, regardless of OUD status, and family income was not a factor distinguishing adults with buprenorphine misuse and OUD from the other examined groups. Thus, perceptions that persons of racial and ethnic minority groups and people living in poverty are more likely to misuse their medication are incorrect. Nevertheless, these factors have been found to be important factors associated with opioid harms and receipt of buprenorphine treatment. Notably, from 2015 to 2017, Black persons aged 25 to 34 years had the largest percentage increase in rates of drug overdose deaths involving any opioids and Hispanic individuals aged 45 to 54 years had the largest percentage increase in overdose death rates involving synthetic opioids,^36^ but White individuals were more likely to receive buprenorphine treatment for OUD.^14,15,37,38^ Furthermore, among adults using prescription opioids, Medicaid beneficiaries and uninsured adults are 2 to 3 times more likely to have OUD than those with private insurance^24^; however, those with private insurance tend to receive buprenorphine treatment for OUD,^14^ and low-income people also face additional financial barriers to buprenorphine treatment.^36,37^ Even among Medicaid enrollees, non-Hispanic Black individuals had less use of medications for OUD than their White counterparts.^38^ These findings, along with evidence that sociodemographic factors do not affect buprenorphine treatment engagement,^39^ underscore the urgency to address economic, health insurance, and racial and ethnic disparities in buprenorphine treatment access.

Researchers have identified that the growth of waivers for clinicians to prescribe buprenorphine is markedly slower in small nonmetropolitan counties than urban counties^8^ and that rural counties are associated with low buprenorphine dispensing.^9^ We found that among adults with buprenorphine use and OUD, residing in nonmetropolitan areas was associated with buprenorphine misuse. Together, these results highlight the importance of strengthening buprenorphine treatment access and treatment quality in rural areas (eg, by expanding and improving access to broadband and other technologies for telehealth services).

The US opioid and suicide crises overlap, because researchers have found that suicide is a silent contributor to opioid overdose deaths^40,41^ and that suicidal ideation before opioid overdose is common.^41,42^ Similarly, we found that among adults with buprenorphine use, 8.2% to 12.3% of adults with OUD (with and without buprenorphine misuse, respectively) and 11.0% of adults with buprenorphine misuse but without OUD reported making a suicide plan in the past year; by contrast, 2.0% of adults with buprenorphine use but without buprenorphine misuse and without OUD planned suicide in the past year. Our multivariable results are consistent with these descriptive findings. Together, these results suggest that both OUD and buprenorphine misuse are associated with suicide risk. Having a suicide plan is considered a psychiatric emergency because it is associated with imminent lethal attempts.^43,44,45^ Thus, for patients using buprenorphine, providing timely and tailored interventions to reduce suicide risk and prevent opioid overdose deaths due to suicidal intent is warranted.

Our multivariable results also suggest that other substance use and use disorders are quite common in adults who misuse buprenorphine, consistent with previous research on correlates of prescription opioid misuse.^24,46,47,48,49,50^ Such co-occurrences are a reminder for clinicians that buprenorphine misuse often co-occurs with use and use disorders of multiple substances.^51^ Because polysubstance use and use disorders are associated with increased risk of overdose and negatively associated with buprenorphine treatment engagement^40^ and retention,^52,53^ early screening and timely interventions for co-occurring substance use and use disorders are critical.

We found that among adults with buprenorphine use, regardless of their OUD status, those receiving treatment for drug use were less likely to misuse buprenorphine than those not receiving drug use treatment. Moreover, adults with OUD and buprenorphine misuse were more likely to report “because I am hooked” as a motivation for their most recent buprenorphine misuse compared with their counterparts with nonbuprenorphine prescription opioid misuse. Because only 43% of US adults with buprenorphine misuse and OUD received drug use treatment in the past year, and because our study and multiple other studies^21,22,23,54^ found self-treatment of craving and withdrawal symptoms as the predominant motivation for using nonprescribed buprenorphine among people with OUD, our results highlight the need for adults with OUD to engage and be retained in good-quality buprenorphine treatment. These findings underscore the importance of future research to improve understanding of strategies that improve treatment access, engagement, and retention. Importantly, the chronic nature of addiction, along with the time needed to stabilize a patient receiving buprenorphine, should preclude administrative discharge of patients from treatment based on detection of misuse. In addition, the prevalence of individuals with buprenorphine use but with neither buprenorphine misuse nor OUD remained stable from 2015 to 2019. This group could include people undergoing management of chronic pain, people receiving long-term treatment with buprenorphine and in recovery for their opioid use disorder for more than 12 months, or both. Future research is needed to continue monitoring related trends among this group while expanding access to buprenorphine-based OUD treatment and developing strategies to reduce buprenorphine misuse.

### Limitations

Our study has several limitations. The NSDUH excludes people experiencing homelessness and not living in shelters or people residing in institutions (eg, incarcerated adults), which could lead to underestimates in drug use and use disorders and suicidality. Because of the cross-sectional nature of NSDUH data, we could not establish temporal or causal relationships. Future studies may examine the specific timing of measures of past-year behaviors (eg, misuse before or during opioid treatment) and related clinical implications. More research is needed to separately assess the misuse measure of buprenorphine, distinguishing use without a prescription (a sign of diversion) from use without following a physician’s instructions to understand related clinical implications. The NSDUH neither assesses pain or pain management nor captures the details of treatment with buprenorphine. In addition, the NSDUH is a self-reported survey and is subject to recall bias. Additionally, future research should examine how changes to buprenorphine prescribing during the COVID-19 pandemic (eg, prescription via telehealth by clinicians who receive waivers^55^ and increasing coverage through emergency Medicaid expansion^56^) affect buprenorphine misuse.

## Conclusions

The prevalence of buprenorphine misuse with OUD had a downward trend among adults with buprenorphine use in the US during the period from 2015 to 2019. Encouragingly, in 2019, nearly three-fourths of US adults reporting past-year buprenorphine use did not misuse their prescribed buprenorphine, and most who misused reported using prescription opioids without having their own prescriptions. Our findings underscore the need to pursue actions that expand access to buprenorphine-based OUD treatment, to develop strategies to monitor and reduce buprenorphine misuse, and to address conditions associated with misuse such as chronic pain, suicide risk, co-occurring mental illness, and polysubstance use.

## Notes

Supplement. **eTable.** Multivariable Multinomial Logistic Regression Factors Associated With Past-Year Opioid Use Disorder and Buprenorphine Misuse Status Among US Adults With Past-Year Buprenorphine Use
