# Supplementary material for: Trends in and Characteristics of Buprenorphine Misuse Among Adults in the US
Source: JAMA Netw Open. 2021 Oct 15;4(10):e2129409. doi: 10.1001/jamanetworkopen.2021.29409 (PMC8520126; doi:10.1001/jamanetworkopen.2021.29409)
Supplement: Supplement. — eTable. Multivariable Multinomial Logistic Regression Factors Associated With Past-Year Opioid Use Disorder and Buprenorphine Misuse Status Among US Adults With Past-Year Buprenorphine Use [file jamanetwopen-e2129409-s001.pdf]

Supplementary Online Content

Han B, Jones CM, Einstein EB, Compton WM. Trends in and characteristics of buprenorphine misuse among adults in the US. *JAMA Netw Open*. 2021;4(10):e2129409. doi:10.1001/jamanetworkopen.2021.29409

eTable. Multivariable Multinomial Logistic Regression Factors Associated With Past-Year Opioid Use Disorder and Buprenorphine Misuse Status Among US Adults With Past-Year Buprenorphine Use

This supplementary material has been provided by the authors to give readers additional information about their work.

© 2021 Han B et al. *JAMA Network Open*.

eTable. Multivariable Multinomial Logistic Regression Factors Associated With Past-Year Opioid Use Disorder and Buprenorphine Misuse Status Among US Adults With Past-Year Buprenorphine Use

	**Adjusted OR (95% CI)**					
Among Adults with Past- Year Buprenorphine Use	**OUD:** Misuse vs. No misuse	**No OUD:** Misuse vs. No misuse	Misuse & OUD vs. No Misuse & No OUD	**Misuse:** OUD vs. No OUD	**No Misuse:** OUD vs. No OUD	Misuse, No OUD vs. No Misuse, OUD
**Age**						
18-23	2.0 (0.84-4.98)	2.0 (0.87-4.78)	1.7 (0.73-4.09)	0.8 (0.35-2.05)	0.8 (0.34-2.12)	**2.4 (1.01-5.81)**
24-34	**2.9 (1.42-5.77)**	**2.1 (1.08-4.22)**	**2.8 (1.41-5.61)**	1.3 (0.62-2.77)	1.0 (0.45-2.13)	**2.2 (1.08-4.40)**
35-49	**2.3 (1.20-4.52)**	1.6 (0.88-2.82)	**2.4 (1.23-4.64)**	1.5 (0.69-3.32)	1.0 (0.44-2.38)	1.5 (0.64-3.68)
≥50+	1.0	1.0	1.0	1.0	1.0	1.0
**Sex**						
Men	0.8 (0.53-1.25)	1.1 (0.69-1.68)	1.1 (0.73-1.68)	1.0 (0.63-1.67)	1.4 (0.92-2.02)	0.8 (0.48-1.31)
Women+	1.0	1.0	1.0	1.0	1.0	1.0
**Race/Ethnicity**						
NH White+	1.0	1.0	1.0	1.0	1.0	1.0
NH Black	1.5 (0.78-2.82)	1.0 (0.36-2.59)	2.2 (0.80-6.23)	2.3 (0.81-6.68)	1.5 (0.49-4.65)	0.6 (0.21-1.91)
Hispanic	0.7 (0.38-1.45)	1.3 (0.64-2.56)	0.9 (0.51-1.61)	0.7 (0.39-1.30)	1.2 (0.63-2.41)	1.0 (0.48-2.25)
NH other	0.9 (0.41-1.87)	1.2 (0.54-2.70)	1.2 (0.53-2.57)	1.0 (0.38-2.48)	1.3 (0.63-2.87)	0.9 (0.38-2.12)
**Family Income**						
<$20,000	1.1 (0.58-1.88)	**1.9 (1.14-3.27)**	1.0 (0.50-1.83)	0.5 (0.24-1.01)	0.9 (0.50-1.65)	**2.1 (1.12-4.04)**
$20,000-$49,999	1.1 (0.62-1.82)	1.3 (0.77-2.29)	0.8 (0.46-1.48)	0.6 (0.34-1.15)	0.8 (0.44-1.37)	1.7 (0.90-3.24)
$50,000-$74,999	2.0 (0.91-4.28)	1.2 (0.62-2.36)	1.0 (0.51-1.83)	0.8 (0.38-1.69)	**0.5 (0.24-0.99)**	**2.5 (1.13-5.30)**
≥$75,000+	1.0	1.0	1.0	1.0	1.0	1.0
**Metropolitan Statistical Area**						
Large metro+	1.0	1.0	1.0	1.0	1.0	1.0
Small metro	1.5 (0.98-2.22)	1.4 (0.86-2.12)	1.0 (0.59-1.53)	0.7 (0.37-1.32)	0.7 (0.42-1.00)	**2.1 (1.15-3.81)**
Non-metro	**1.8 (1.04-2.99)**	1.4 (0.85-2.37)	1.0 (0.60-1.51)	0.7 (0.40-1.13)	**0.5 (0.31-0.94)**	**2.6 (1.52-4.59)**
**Suicide Plan**						
Yes	0.5 (0.23-1.14)	**4.1 (1.69-9.83)**	**2.5 (1.06-5.76)**	0.6 (0.25-1.46)	**4.8 (2.45-9.39)**	0.9 (0.36-2.03)
No+	1.0	1.0	1.0	1.0	1.0	1.0
**Tobacco Use & Disorder**						
PM nicotine dependence (ND)	1.2 (0.63-2.44)	1.4 (0.75-2.61)	**3.0 (1.47-6.28)**	2.2 (0.99-4.67)	**2.5 (1.32-4.57)**	0.6 (0.27-1.22)
PY tobacco use, no PM ND	0.9 (0.46-1.79)	1.0 (0.56-1.95)	1.8 (0.92-3.52)	1.7 (0.83-3.62)	**2.0 (1.06-3.70)**	0.5 (0.24-1.16)
No PY tobacco use+	1.0	1.0	1.0	1.0	1.0	1.0
**Alcohol Use & Disorder**						
PY alcohol use disorder	1.6 (0.94-2.75)	**1.8 (1.02-3.22)**	1.0 (0.59-1.73)	0.6 (0.29-1.07)	0.6 (0.34-1.15)	**2.9 (1.61-5.16)**
PY alcohol use, no disorder	1.5 (0.97-2.33)	**1.7 (1.06-2.85)**	1.2 (0.76-1.74)	0.7 (0.38-1.14)	0.8 (0.49-1.19)	**2.3 (1.25-4.12)**
No PY alcohol use +	1.0	1.0	1.0	1.0	1.0	1.0

© 2021 Han B et al. *JAMA Network Open*.

Among Adults with Past-Year Buprenorphine Use	**OUD:** Misuse vs. No misuse	**No OUD:** Misuse vs. No misuse	Misuse & OUD vs. No Misuse & No OUD	**Misuse:** OUD vs. No OUD	**No Misuse:** OUD vs. No OUD	Misuse, No OUD vs. No Misuse, OUD
**Cannabis Use & Disorder**						
PY cannabis use disorder	1.4 (0.65-3.05)	0.8 (0.38-1.49)	1.9 (0.97-3.56)	**2.5 (1.18-5.19)**	1.3 (0.66-2.63)	0.6 (0.26-1.26)
PY cannabis use, but no disorder	1.2 (0.78-1.90)	**1.5 (1.02-2.24)**	**1.6 (1.05-2.55)**	1.1 (0.65-1.82)	1.4 (0.86-2.10)	1.1 (0.64-1.95)
No PY cannabis use+	1.0	1.0	1.0	1.0	1.0	1.0
**Cocaine Use & Disorder**						
PY use or disorder	**2.3 (1.09-4.71)**	**4.0 (2.35-6.92)**	**9.8 (4.81-20.10)**	**2.4 (1.30-4.59)**	**4.3 (1.81-10.38)**	0.9 (0.41-2.10)
Lifetime use, no PY use	**1.9 (1.02-3.59)**	**2.1 (1.37-3.28)**	**3.7 (2.14-6.45)**	**1.8 (1.03-3.00)**	1.9 (0.97-3.89)	1.1 (0.51-2.36)
Never use+	1.0	1.0	1.0	1.0	1.0	1.0
**Rx Sed./Tranq. Misuse & Disorder**						
PY misuse & disorder	0.9 (0.46-1.66)	0.9 (0.57-1.50)	**1.7 (1.09-2.72)**	**1.9 (1.13-3.03)**	2.0 (1.00-3.87)	0.5 (0.22-1.03)
PY use, no misuse	**0.3 (0.16-0.64)**	**0.3 (0.19-0.47)**	**0.4 (0.23-0.71)**	1.4 (0.83-2.22)	1.3 (0.62-2.63)	**0.2 (0.11-0.49)**
Lifetime use, no PY use	0.6 (0.25-1.34)	0.7 (0.34-1.45)	0.5 (0.28-1.00)	0.8 (0.35-1.61)	0.9 (0.45-1.86)	0.8 (0.29-2.01)
Never use+	1.0	1.0	1.0	1.0	1.0	1.0
**Rx. Stimulant Misuse & Disorder**						
PY use disorder	**3.9 (1.34-11.23)**	3.3 (0.91-12.35)	**4.0 (1.37-11.60)**	1.2 (0.43-3.27)	1.0 (0.26-4.04)	3.3 (0.78-13.62)
PY misuse, no disorder	1.6 (1.00-2.62)	1.2 (0.68-2.16)	0.7 (0.36-1.38)	0.6 (0.32-1.04)	**0.4 (0.21-0.88)**	**2.8 (1.62-4.81)**
No PY misuse +	1.0	1.0	1.0	1.0	1.0	1.0
**PY Hydrocodone Product Misuse**						
Yes	0.8 (0.51-1.30)	**4.3 (2.65-7.08)**	**4.9 (2.97-8.16)**	1.1 (0.74-1.75)	**6.9 (3.10-11.73)**	0.7 (0.39-1.32)
No+	1.0	1.0	1.0	1.0	1.0	1.0
**PY Oxycodone Product Misuse**						
Yes	**1.9 (1.18-3.05)**	**3.9 (2.31-6.46)**	**10.0 (5.77-16.23)**	**2.5 (1.49-4.21)**	**5.1 (2.80-9.29)**	0.8 (0.38-1.50)
No+	1.0	1.0	1.0	1.0	1.0	1.0
**PY Substance Use Treatment**						
Drug only	**0.4 (0.27-0.67)**	**0.4 (0.21-0.58)**	**1.7 (1.05-2.82)**	**4.9 (2.79-8.67)**	**4.0 (2.60-6.17)**	**0.1 (0.05-0.15)**
Both alcohol and drug	1.0 (0.56-1.90)	0.7 (0.31-1.64)	**2.6 (1.40-4.82)**	**3.6 (1.84-7.10)**	**2.5 (1.17-5.43)**	**0.3 (0.14-0.58)**
None+	1.0	1.0	1.0	1.0	1.0	1.0

Data source: 2469 respondents of 2015-2019 National Surveys on Drug Use and Health data. Misuse=buprenorphine misuse; OUD=opioid use disorder; PY=past year; PM=past month; Rx=prescription; Sed./Tranq=sedative or tranquilizer; Each **bolded** estimate is statistically significantly (*P*<0.05) different from the estimate of the reference group (with + sign). Other variables, which were presented in Table 2, but not in Table 3, were not significantly associated with the outcomes and were removed from this final multinomial logistic regression model. Age, sex, and race/ethnicity remained in the final model regardless of their statistical significance.

© 2021 Han B et al. *JAMA Network Open*.
